# Re‐humanizing anatomy? Learning through human body donors in physiotherapy education

**DOI:** 10.1002/ase.70322

**Published:** 2026-08-13

**Authors:** Robert Parish, Chris Carter, John Stephens, Debs Patten

**Affiliations:** ^1^ Faculty of Health Science & Wellbeing University of Sunderland Sunderland UK

**Keywords:** body donor prosection, learning anatomy, person‐centered, physiotherapy, professional identity

## Abstract

Anatomical knowledge underpins clinical skills and professional identity in physiotherapy. However, dedicated teaching has declined in recent years. Body donor‐based learning, though common in medical education, is rarely used in physiotherapy. Its integration may enhance learning through embodied, haptic experiences, and greater physical and emotional engagement with anatomical structures. A qualitative methodology grounded in phenomenology and hermeneutics was employed, using Interpretative Phenomenological Analysis (IPA) to explore how final‐year BSc (Hons) Physiotherapy students made sense of their experiences of learning anatomy through haptic interaction with body donors facilitated by a supersoft‐fix preservation technique. This study provides a novel perspective of the impact of learning anatomy through body donors and prosections for physiotherapy students. While learning anatomy with body donors affords students deep and embodied haptic learning opportunities, there are emotional aspects, which physiotherapy educators must consider. These emotional aspects can influence how students perceive the humanity or non‐humanity of body donors. Soft‐fixation embalming techniques permit students to mobilize joints and observe dynamic functional movement, which enhances students' anatomical understanding. However, educators must consider the variable and complex nature of how physiotherapy students view and interact with body donors and the potential influence on these experiences toward developing their professional identity. Re‐humanizing anatomy is a dynamic learning process where students describe moving within a *“Body Donor Learning Continuum”* (BDLC) where, over time and through reflection, they experience a spectrum of negative to positive emotions and reactions.

## INTRODUCTION

Anatomical knowledge provides a foundation for healthcare professionals,^1^ including physiotherapy where it functions to build key skills that underpin the scope of practice and identity.^2^ The knowledge and understanding of anatomy are an integral component of physiotherapy undergraduate education within the United Kingdom (UK).^3^

Over recent years, an anatomy syllabus void has been identified within physiotherapy.^2^ Potential reasons include a decrease in allocated curriculum hours, driven by the continually expanding scope of professional practice.^4^ To meet this challenge, anatomical education in physiotherapy must evolve and be underpinned by robust pedagogy which recognizes the importance of physical fidelity and embodied, haptic learning within practical teaching contexts.^4^, ^5^ Although teaching with body donors has been a cornerstone of medical education for centuries, few UK Higher Education Institutions currently offer such experiences beyond medicine and dentistry.^2^ Currently, a handful of UK universities state they offer BSc or MSc physiotherapy students the opportunity to work with embalmed body donors and/or prosections as part of their training. This study explores final year BSc (Hons) Physiotherapy students' perceptions of anatomy learning through haptic interaction with body donors facilitated by a supersoft‐fix preservation technique.

As well as the development of anatomical knowledge, body donor education has the potential to make a meaningful contribution to professional identity, cultivating essential values such as respect, dignity, and compassion.^6^, ^7^, ^8^ Early exposure to body donor teaching has been shown to be associated with deeper ethical reflection and a heightened sense of professional responsibility.^9^, ^10^, ^11^ Engaging with mortality in this context can foster a sense of gratitude and ethical awareness, contributing to the “hidden curriculum” that supports students' broader professional development.^12^, ^13^, ^14^ Linking anatomical learning to future clinical roles enhances students' ability to humanize patients and appreciate the person behind the pathology.^15^, ^16^, ^17^

The Anatomy Centre (AC) at the University of Sunderland (UoS), constructed to support its new medical school, was opened in April 2022.^18^ It represents a distinctive and innovative facility encouraging haptic learning experiences to a range of healthcare programs, including physiotherapy. This novel approach is underpinned by two rationales: it fosters a patient‐centered perspective and leverages an innovative supersoft‐fixation preservation method for body donor anatomy.^19^ Supersoft‐fixation techniques have been shown to be safer, more sustainable, and more cost‐effective than traditional embalming. They also enable the joint mobilization and dynamic visualization of functional movement, which cannot be achieved with traditional embalming.^20^, ^21^ This has the potential to enhance students’ anatomical understanding and to support the development of professional skills and attributes essential to physiotherapy practice.^16^

While the educational value of body donor teaching has been widely explored in medicine and nursing education,^6^, ^10^, ^22^, ^23^ there remains limited research on its role in physiotherapy education.^15^ Little is known about how undergraduate physiotherapy students engage with, interpret, and experience body donor anatomy and its contributions to their learning.^4^ The study sought to answer the following: How do physiotherapy students make sense of their haptic interactions facilitated with supersoft‐fixed body donors and prosections during the formation of their professional identity?

## METHODOLOGY AND METHODS

The study was framed within an Interpretative Phenomenological Analysis (IPA) approach to explore students' lived experiences.^24^ IPA is grounded in phenomenology and hermeneutics, focusing on participants' perspectives and their processes of making sense of their lived experiences.^25^

Purposive sampling was employed^26^ to recruit a homogenous sample (*n* = 6) of final‐year BSc (Hons) Physiotherapy students from the University of Sunderland who had prior exposure to anatomy sessions involving supersoft‐fix preserved body donor anatomy.

The inclusion criteria included third year physiotherapy students who had attended all body donor anatomy sessions. Students from other years or those without full attendance were excluded. This was to enhance sample homogeneity by ensuring participants shared comparable learning experienced relevant to the study aim. IPA prioritizes depth over breadth by encouraging a small, purposively selected, and relatively homogeneous sample to support a detailed, idiographic analysis of lived experiences.

The primary researcher (RP) occupied an emic position within the educational context being studied. Reflexive awareness of this positionality was maintained through analytical memo‐writing and regular peer debriefing with the research team. These processes enabled critical examination of assumptions and helped ensure that the analysis remained grounded in participants' interpretations rather than preconceived beliefs.

Given the emic position of the primary researcher (RP), steps were taken to minimize feelings of coercion and reduce perceived power imbalances, to allow participants to respond freely. Participation was voluntary; recruitment was managed through a gatekeeper (JS) instead of direct researcher invitation.

Participant information stated that participation or non‐participation would not influence academic assessment or program progression. The interviewer and primary researcher (RP) held no teaching or evaluative role over participants.

Ethical approval was granted through the University Research Ethics Committee (UREC Serial Number 029473). Informed consent was obtained, confidentiality maintained through two‐character codes, with only RP aware of identities,^27^ well‐being information was shared during debrief.

Two pilot interviews tested and refined the interview schedule and were excluded from analysis.^28^ Individual semi‐structured interviews were conducted by RP, audio‐recorded, and supported by field notes to capture non‐verbal cues.^29^, ^30^ Guided by literature, questions explored responses to anatomical education and professional identity development. Interviews lasted 42–57 min, were transcribed verbatim, and returned to participants for verification.^31^, ^32^ Recordings were deleted after transcription, and transcripts stored securely in password‐protected cloud storage.

Data analysis followed the six‐step IPA process described by Smith et al.^31^ (Figure 1), cyclical bracketing occurred throughout. Each transcript was read repeatedly to develop familiarity with the data, and descriptive, line‐by‐line exploratory notes were recorded. These notes reflected each participant's psychological and experiential context and were interpreted through the hermeneutic circle. Consistent with IPA's double hermeneutic nature, analysis involved continual cross‐referencing of statements to ensure grounding in the data.

**FIGURE 1:**
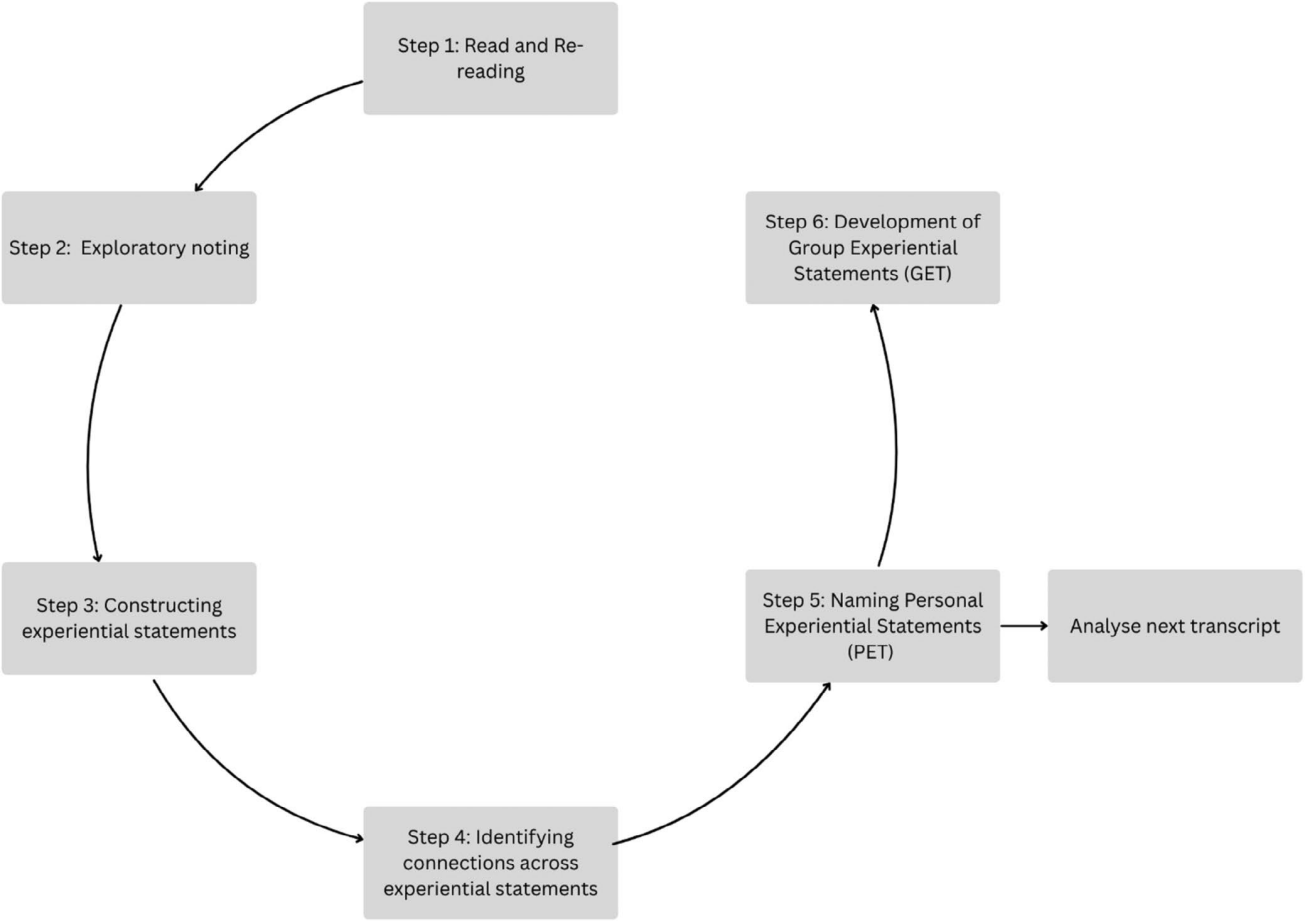
Analysis process adapted from Smith et al.^31^

This process was conducted for each transcript. Exploratory notes were clustered into experiential statements, with incongruent or unsupported themes excluded.^31^ Following complete analysis of transcripts by RP, a transcript was discussed with supervisor (CC) and experiential statement clusters were refined collaboratively. These experiential statement clusters then developed into Personal Experiential Themes (PETs). PETs were examined for patterns and similarities across participants and organized into Group Experiential Themes (GETs). These GETs represent shared experiential patterns across participants while preserving the idiographic insights derived from individual accounts.

The thematic structure, including participants' GETs, PETs, and supporting experiential statements, is presented in Table 1.

**TABLE 1:** Thematic table with participants' GETs, PETs, and experiential statements.

GET	PET	Experiential statements
Humanization vs. dehumanization	From body to person	Acknowledgement of the previous life of the cadavers
		Identifying cadavers as people
		Giving the cadavers life stories
		Contemplation about the life experience of cadavers
		Assigned personhood
	From person to machine	Description of body as a machine
		De‐personalization through objectification
		Body exists to support the brain
		Viewing the body as a vessel
		Segmentation of the body into its constituents
Navigating emotional change	Negative emotional response	Feelings of disgust when seeing the dead
		Touching the cold cadavers was nauseating
		Upsetting scenario
		Pre‐exposure and exposure anxiety
		Viewing the cadaver shocked them
	Dissociation	Feelings of zoning out
		Out of body feelings
		Visual abnormalities following seeing the cadavers when in the lab
		Contemplating their own mortality
	Positive reflection	Feeling positive about the experience when reflecting
		Improvements in emotions after the first time
		Development of appreciation and gratitude
		Happiness about completing the experience
		Better understanding about the role of cadavers in their education

## FINDINGS AND DISCUSSION

Following analysis of the transcripts, two GETs were expressed, “Humanization vs. Dehumanization” and “Traversing emotional change” (see Figure 2, below). The themes are interconnected and independent, with students describing changing perspectives and emotions toward body donors, with proximity to the body donors contributing heavily to this. The PETs relating to Humanization and Dehumanization will be initially discussed to frame a continuum of body donor learning articulated at Figure 3.

**FIGURE 2:**
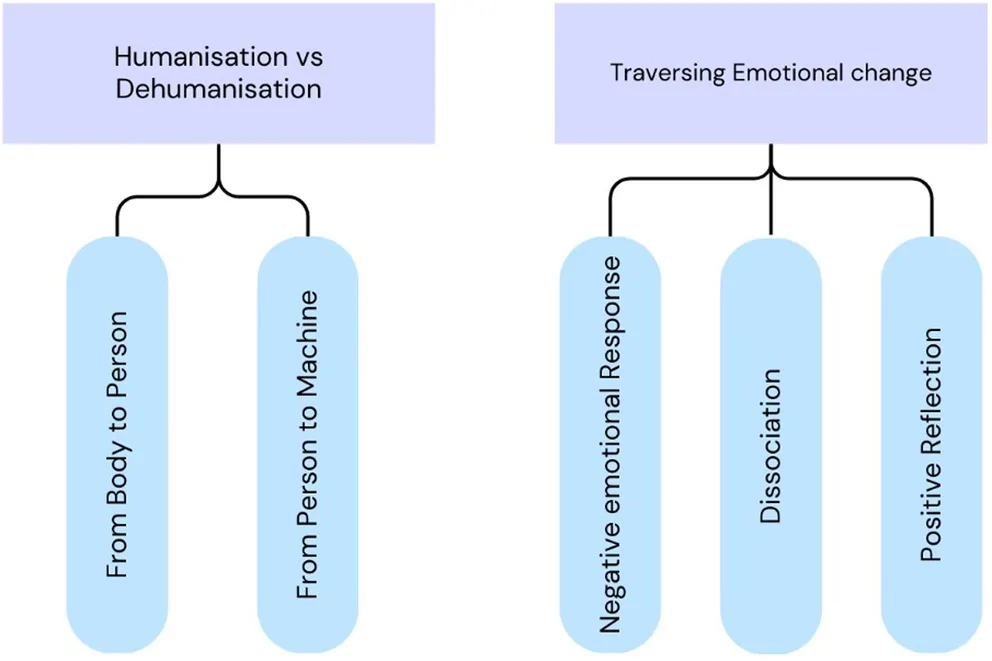
Relationship between the personal experiential and group experienced themes.

**FIGURE 3:**
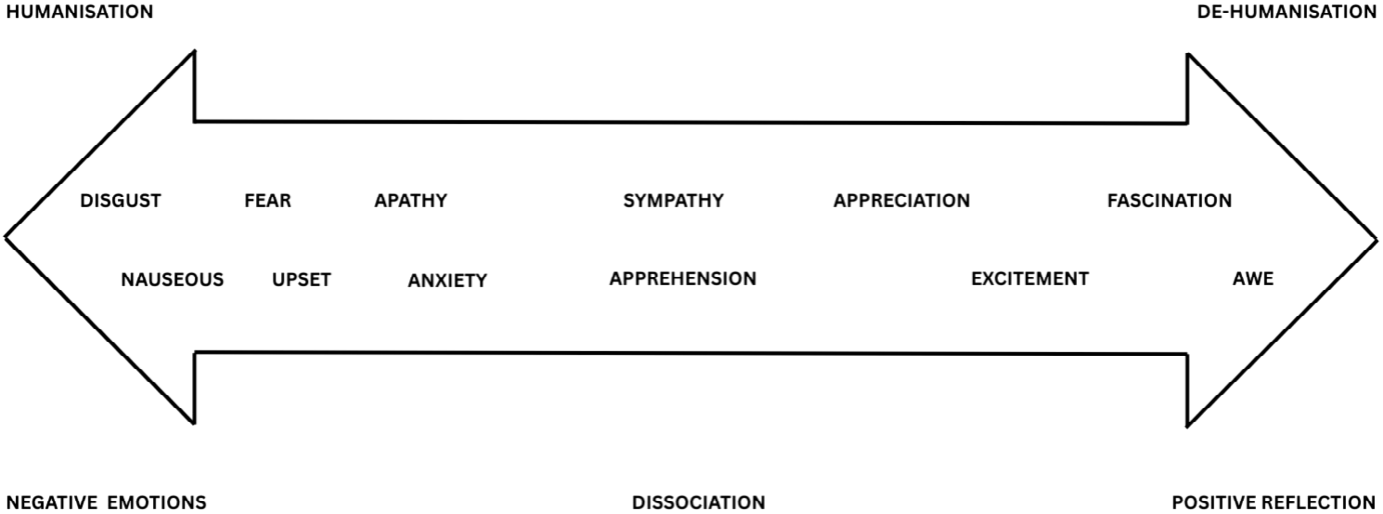
The Body Donor Learning Continuum (BDLC).

### Humanization vs. dehumanization

Although body donor anatomy is widely acknowledged as an effective means of acquiring anatomical knowledge, the interaction with body donors can be emotionally and psychologically demanding and lead to significant psychological stress.^33^ Students are often required to reconcile the educational experience with the reality of human mortality, leading to varied emotional reactions and perceptions.^33^, ^34^ These responses may influence how students engage with body donors—viewing them alternately as patients, teachers, specimens, or deceased individuals.^35^, ^36^

There was kind of a specimen who had fingernails painted, erm, I was like, Oh, this is actually a person now which was hard to comprehend. A1

This moment illustrates how small personal markers of identity disrupted the student's ability to maintain emotional distance. The presence of painted fingernails served as a reminder of the donor's previous life, prompting a temporary shift toward humanization. The way students perceived body donors was strongly influenced by the extent to which the body is humanized. When personal detail such as gender, age and occupation are shared, this can foster feelings of gratitude and reverence.^37^ Conceptualizing body donors as “first patients” can help students adopt a respectful, professional stance, limiting emotional distress and promoting empathy.^15^, ^38^, ^39^, ^40^ Conversely, for some students, over‐identification with the “human referents” of the body donor may hinder learning, prompting them to depersonalize the body as a self‐protective measure.^41^ It is this ambiguity in conceptualizing the donor, as something like an object, as well as like a human, that manifests some of the most challenging ethical and conceptual issues surrounding body donor anatomy education.^16^

Thus, this GET comprises the psychological reasoning students navigate when presented with such challenges. The multifaceted perceptual change of viewing body donors as human and non‐human objects was transient and causes cognitive dissonance spoken to by two core PETs: “From body to person” and “From Person to Machine.”

### From body to person

Throughout the interviews, students articulated the sense of realization of the personhood and humanity of the body donors. This distinction was placed on visual features with categorization on the individuality and personality or the shared humanity of the body donors.

It's one thing to know that it's a human body, but when you're standing in front of it, you're reminded that this was a person who had their own life and experiences. D4

Students acknowledged the body donor as a “human body” prior to the experience but when “standing in front” of the body donor the realization of personhood becomes present, suggesting they did not expect to personalize the body donor. Lazarus, Douglas and Stephens^42^ share similar findings of student's experiencing significant unexpected realization of the personhood of the body donor and mention being “reminded” of the lived life of the body donor.

The realization of personhood was evident in other students' narratives, noting certain features as the cause of their realization.

I think seeing the small things like scars on the whole body made it a bit personal, like you can kind of see their story. It makes you think about the life. E5

The presence of physical marks such as “scars” and painted nails (see previous) are noted as catalysts for perceptual change, from body donor to person. The comment “see their story” illustrates a shift from superficial acknowledgement of the previous life of the body donor causing contemplation of questions surrounding their lived experiences. Although students consciously understand the individuality of the body donors, this is challenged throughout the interaction with personal features causing a visceral reaction to the sonder of the body donors; finding life in something with no life.^43^, ^44^ Seeing such features as nail polish and tattoos often causes an innate reaction to assign personhood to the body donor. Assigning personhood was not only present in full body donors but also was assigned to single organs.

When I was holding the brain, I felt that I was holding the person's life and memories. It was really personal; I needed to respect it. F6

This moment captures a transition from body to person, as the student begins to encounter the organ, the brain, not simply as an anatomical structure but as a site of lived experience. In this way, organs such as the brain and heart invite more humanistic and meaningful connections with body donors, foregrounding their previous lives and personhood.^45^, ^46^ Personal and cultural beliefs dictate the perceptual importance of the brain. In Western cultures, like the United Kingdom, the brain is often seen as the place of the soul and the essence of the person, leading many students to associate it with the person rather than their body.^42^, ^47^

Students commonly apply personal features to body donors to build a more personalized account and not to view them solely as an anatomical tool. The identification of posthumous personality for deceased humans is an interesting finding not specifically identified previously; however, this does relate to seeing a person as a whole being and not a fragmentation of parts. Practices such as personalizing body donors can help students employ a more person‐centered approach to clinical practice.^15^

### From person to machine

To help with learning I started to look at them as machines rather than people, it helped me focus on learning anatomy instead of the human bit. F6

The student explicitly states a conscious shift from people to machines, in a deliberate effort to facilitate learning as they found the “human bit” a distraction or impediment to anatomical study. By reframing human body donors to non‐human forms like machines, students limit the psychological distraction of death and personhood and focus more on learning.^48^ The quote directly contradicts students' earlier statements about holding the brain and “holding the person's life and memories,” this conflict between the recognition of “life and memories” and the subsequent adoption of the “machine” metaphor underscores an emotional and cognitive shift due the demands of body donor anatomy education.

I looked at the lungs and heart as just there to keep the brain alive, like a machine. it helped dehumanised them, it helped me learn. B2

The student reduced the organs to parts of a machine with a sole purpose of keeping “the brain alive,” which reflects a simplified view of highly complex organs. Students dehumanize body donors to rationalize their actions in response to cognitive dissonance surrounding the experience allowing students to focus on learning.^49^

I tried kind of just looked at them as objects, like slabs of meat or anatomy models. It made it non‐human in my head, I lost the emotions for them. C3

The terms “objects,” “slabs of meat,” and “models” indicate a distancing from the human aspect through objectification, with the phrase “I lost emotions” alluding this being a coping mechanism. Objectifying body donors seemed to be a mechanism indicating a purposeful approach to dehumanizing body donors for various reasons but all with the goal to focus on learning and controlling emotions. Objectification is typically used to reduce the significance of the body donors to that of any other object.^50^ This process is not malicious but is an attempt of gaining control and power in a situation, which is highly emotional and traumatic.^51^

Whether students view a body donor as a formerly living human or as an impersonal object is not a simple dichotomy, and that the body donor is “an ever‐changing collage of attributes and configurations which are often in conflict and mired in ambiguity”.^52^ ^(p97)^ The conflict of the donor as a human, “something like me” as well as “an object,” something “alien, strange, not‐me”^52^ ^(p101)^ manifests as some of the most challenging ethical and conceptual issues surrounding body donor anatomy education. A linguistic turn that perhaps further reflects this conflict is represented in the use of the more contemporary term “body donors” as opposed to the term “cadavers” in the past.

The interconnectivity of PETs demonstrates the interplay of humanization and dehumanization techniques in response using body donors to learn anatomy in undergraduate physiotherapy education. When students are presented with this impasse, they can latently switch perspectives between a human being (emotional perspective) and a learning tool (rational perspective).^41^

The tactile realism afforded by supersoft‐fix preservation may intensify students' emotional engagement with donor tissue compared to traditional formalin‐preserved specimens. This heightened haptic fidelity likely contributes to the oscillation between humanization and dehumanization.

### Traversing emotional change

The processes of reframing emotional and rational perspectives of body donors is present in the findings and surrounding literature.^15^, ^53^ Students' perceptions are not static but exist in a fluid state, which is directed by emotions and cognitive focus.^15^ The changes in emotions experienced were continually referenced in student accounts as being primary contributors to perceptual change in body donors. This process of the fluid state between Dehumanization and Humanization is captured in the second GET, “Traversing Emotional Change,” represented by the “Body Donor Learning Continuum” (BDLC) (Figure 3, below).

The BDLC is proposed as a conceptual model that captures the dynamic emotional and cognitive processes experienced by physiotherapy students when learning anatomy through body donor interaction. Rather than representing a fixed progression, the continuum illustrates how students may move fluidly between humanization and dehumanization as they negotiate emotional responses, sensory experiences, and cognitive coping strategies. Recognizing this oscillation provides a useful pedagogical lens for educators seeking to support students in navigating the emotional complexity of body donor anatomy education.

Traversing emotional change therefore reflects the adaptive emotional responses of students while working with body donors and how both personal beliefs and experiential learning shape these responses over time. Within this present study, these emotional shifts were commonly described through three distinctive PETs: “Negative emotional response,” “Dissociation,” and “Positive reflection.”

### Negative emotional response

Five students held a shared PET of experiencing negative emotions following initial exposure to body donors. These negative emotions were linked with the act of dehumanizing the body donors, cited below.

I felt anxious before we seen them for the first time. When I seen them I felt quite emotional as it's a dead person. I started looking at them as objects and that helped. C3

Pre‐exposure anxiety was evident with the viewing of the body donor triggering a strong emotional reaction, rooted in the recognition of death. Assigning personhood to body donors can disrupt students' abilities to view body donors objectively, resulting in more negative emotional responses.^54^ Humanizing body donors is intrinsically related to negative emotions and dehumanization helps control these emotions.^55^

Within this study, negative emotions presented uniquely for everyone with a variety of psychological and physical presentations.

I was in a state of shock I think. D4

I felt sad for them, I was upset. F6

Others explained a more psychophysical manifestation of these emotions.

When I first walked in, the smell and sight of the cadaver hit me, I turned away. When I touched it I felt sick, it was disgusting. A1

The immediate turning away indicates a desire to distance oneself from the body donor. Turning reduces the sensory load helping manage the impact. Humans naturally retract from the dead, that stems from feelings of disgust but this reaction is challenged in body donor anatomy education.^56^ Feelings of disgust are common among students encountering body donors for the first time and is considered a normal natural response to viewing dissected body donors.^43^ Emotional responses to handling donor tissue may stem from deeper psychological mechanisms.^57^ These responses can serve to protect individuals from disruptions to their sense of identity, internal order, and psychological equilibrium.^58^ The concept of psychological protection was repeatedly described throughout the interviews in the form of dissociation.

### Dissociation

All students presented dissociative thought processes and negative emotions when working in close proximity with the body donors. Various manifestations of dissociation were reported, with “zoning out,” “feeling numb,” “time stopped,” and visual anomalies being common.

When I first seen it, I just zoned out. I‐I wasn't in the room for a bit. I had never seen a dead person and I don't think I really knew what was going on, my vision went foggy and my mind just went quiet. F6

It was the first time seeing a body, I saw the whole man and felt weird, as if I was in a different place. My brain just got up and walked away [Laugh]. A1

Initial encounters with body donors can impose significant stress and trigger profound psychological responses as people are confronted with mortality. The expressions “zoned‐out” and “My brain got up and walked away” indicate a significant feeling of situational dissociation and indicate a pivotal subjective shift in the consciousness of the students as they temporarily detached from their surroundings. Students often dissociate and detach from body donors to control and cope with negative emotions experienced during body donor anatomy education.^41^

The use of laughter as noted in one account adds a layer of complexity to the quote, it suggests this student attempts to minimize the significance or lighten the experience, perhaps as a coping mechanism. Laughter can serve as a form of stress release in the face of uncertainty and absurdity and serves as a coping mechanism in a situation where death is frontmost, allowing for positive reframing and reinterpretation of the stressor.^59^, ^60^ Dissociation was reported alongside feelings of anxiety.

When I acknowledged that I would be there one day, like I would be dead I had this out of body feeling. I felt weird and anxious about that I will die; I couldn't focus on learning. C3

The “out of body feeling” with an associated anxiety at the personal realization of death has been cited previously.^10^, ^61^ Students may find it difficult to focus on (subject) learning due to dissociation and anxiety as they can begin to question their own mortality and struggle to reorient back to learning.^62^ Conversely, repeated exposure to body donors can result in increased positive perceptions and feelings of appreciation and gratitude to the body donor^61^ through positive reflection.

### Positive reflection

Engaging in reflection on body donor anatomy education through repeated exposure can prompt contemplation of mortality and foster a more constructive understanding of life and death.^63^

I‐I think that my emotions were all over the place on the first visit, but this became less so in the last session. D4

During the first visit I was anxious but after the first visit I felt a lot more appreciation for the body donors, a lot of respect. B2

The reduced emotional turmoil described suggested a process of becoming accustomed and adapting to the environment of the Anatomy laboratory, acknowledging the dynamic emotional response allowing adjustment over time.

On the third visit I was rather excited and curious before and in the session. I think after seeing them on the first and second time I had already gotten used to it, the stories I gave them became part of my appreciation. I had chances to talk to others and reflect myself on the experience, after this I was appreciative of the experience and happy I did it. I think it helps with how I look at patients, I am more aware of importance of their life and experiences. E5

Humanizing the body donors with stories and associated personal features, utilizing self and peer reflection to subsequently nurture positive emotions, beliefs and awareness of the role of body donors can help establish appreciation and gratitude toward the time spent learning with body donors.^15^, ^64^ This is a stark contrast to previous negative and dissociative feelings described with initial contact where students adopted objectification as a coping strategy.

I felt anxious before we seen them for the first time. When I seen them I felt quite emotional as it's a dead person. I started looking at them as objects and that helped. C3

Reflective capacity is arguably essential for all student healthcare professionals, including physiotherapists, in support of emotional preparedness for clinical practice.^12^, ^13^, ^14^ Reflective activity may engender a sense of appreciation and respect toward body donors, contributing to the development of professional identity and shaping students' approach to patient care.^15^, ^16^, ^17^

### Strengths and limitations

The research and associated methodology has enabled an in‐depth exploration of physiotherapy students' lived experience of haptic learning in anatomy using body donors embalmed using a supersoft fixative.

Due to the small sample size, there is no intention to offer generalization but instead to provide a foundation for any future larger studies in this area of educational research as well as further exploration of the emotional responses and how students' personal beliefs and values may influence perceptions of body donor learning.

Operationally, a feature of the study was as a single researcher, student project involving student peers, which holds strengths and limitations associated with insider research.^65^ In an attempt to mitigate associated bias, a process of cyclical bracketing was used during data analysis.^66^ Following each interview, the primary researcher (RP) recorded reflexive notes in a reflective journal. These reflections were revisited during subsequent analytic stages to ensure that interpretations remained grounded in participants' accounts. Finally, throughout the study, regular supervisory meetings supported ongoing reflexive dialogue and critical reflection on the research process and the researcher's positioning.

## CONCLUSION

This study explores physiotherapy students' perceptions and experiences of learning anatomy with body donors. The tactile realism afforded by supersoft‐fix preserved body donor prosections enabled embodied haptic learning, but also intensified emotional engagement, shaping how students perceived the humanity or non‐humanity of body donors. While learning anatomy with body donors affords students deep and embodied haptic learning opportunities, there are emotional aspects, which physiotherapy educators must consider.

Learners can visualize and mobilize tissues to deepen their understanding of anatomy and inform their clinical practice but being exposed to and/or being in the company of a deceased person/ persons also imposes significant psychological stress on students. This stress can influence how students perceive the humanity or non‐humanity of body donors.

The findings of this study allude to a cognitive dissonance between the perceived humanity of body donors and the students' emotional state. Student also describe moving within a “Body donor learning continuum” (BDLC) where, over time and through reflection, they experience a spectrum of negative to positive emotions and reactions.

The findings reveal two important aspects for educators considering introducing body donor anatomy into their programs: the variable and complex nature of how physiotherapy students view body donors when studying anatomy and the potential influence of these experiences toward developing professional identity for physiotherapy students. The BDLC therefore offers a conceptual framework that may assist anatomy educators in anticipating and supporting students' emotional and professional development during body donor‐based learning. This study explores the perceptions of physiotherapy students only, but the findings here may be transferable to other programs where learners also experience body donor anatomy.

Recommendations include scheduling class time for debrief, providing space for conversation and purposeful reflection. Structured staged debriefing, including early exposure and follow‐up reflective opportunities, is proposed. It is proposed such recommendations could support students who are struggling and help them transition toward the most positive end of the BDLC where appreciation and humanization are at the highest levels. Moreover, exemplifying a supportive debriefing process for students has potential implications for navigating emotional situations within clinical practice.

It is proposed such recommendations could support students who are struggling and help them transition toward the most positive end of the BDLC where appreciation and humanization are at the highest levels. Moreover, exemplifying a supportive debriefing process for students has potential implications for navigating emotional situations within clinical practice.

## AUTHOR CONTRIBUTIONS

**Robert Parish:** Conceptualization; investigation; writing – original draft; methodology; formal analysis; visualization; project administration; resources; data curation; writing – review and editing. **Chris Carter:** Writing – review and editing; supervision; resources. **Debs** **Patten:** Writing – review and editing; supervision; resources. **John Stephens:** Writing – review and editing; supervision; resources.

## FUNDING INFORMATION

This research did not receive any specific grant from funding agencies in the public, commercial, or not‐for‐profit sectors.

## CONFLICT OF INTEREST STATEMENT

The authors declare no competing interests.

## ETHICS APPROVAL

The study was approved by the Research Ethics Committee, University of Sunderland, with approval reference number 029473. All participants gave written informed consent before data collection began.

## USE OF GENERATIVE AI

The authors confirm that no artificial intelligence (AI) tools or automated technologies were used in the conception, design, analysis, or writing of this manuscript. All content presented is the original intellectual work of the author(s), who take full responsibility for its accuracy and integrity.

## Data Availability

The data that support the findings of this study are not publicly available due to ethical and privacy restrictions and are not available for sharing.
